# Effect of Irrigants on the Push-Out Bond Strength of Two Bioceramic Root Repair Materials

**DOI:** 10.3390/ma12121921

**Published:** 2019-06-14

**Authors:** Ruaa A. Alamoudi, Sawsan T. Abu Zeid

**Affiliations:** 1 Endodontic Department, Faculty of Dentistry, King Abdulaziz University, Jeddah 22252, Saudi Arabia; sawsanabuzeid55@hotmil.com; 2 Endodontic Department, Faculty of Dentistry, Cairo University, Giza 12345, Egypt

**Keywords:** compressive strength, EndoSequence root repair material, root canal irrigants

## Abstract

The purpose of this study was to compare different irrigants’ effect on two EndoSequence root repair materials’ push-out bond strength. Sixty root slices were filled either with EndoSequence premixed fast-set putty or regular-set paste, and then immersed either in sodium hypochlorite, chlorhexidine gluconate, or saline (as control) for 30 min, after which the slices were subjected to the push-out test. The surface structures were evaluated with Scanning Electron Microscopy and Fourier Transform Infrared. Fast-set putty exhibited greater displacement resistance when immersed in saline and subjected to adhesive failure mode, while regular-set paste showed greater resistance when immersed in chlorhexidine and subjected to cohesive failure mode. Infrared analysis showed changes in organic filler, and carbonate and phosphate bands after using irrigants. The lowest carbonate/phosphate ratio was found for chlorhexidine in both materials. Therefore, sodium hypochlorite reduced EndoSequence root repair materials’ displacement resistance markedly.

## 1. Introduction

Furcation perforation is a communication between the root canal system and the supporting tissues of the teeth. Management of furcation perforations using root repair material poses a challenge for clinicians in efforts to prevent complicated endodontic–periodontal lesions [1,2].

It is ideal for endodontic root repair materials’ to be antibacterial, biocompatible, radiopaque, dimensionally stable, easy to manipulate, and unaffected by blood contamination. It also is desirable for this material to induce or conduct bone deposition, provide a good seal against bacteria and fluids, set in a wet environment, and have sufficient compressive strength and hardness [3,4,5].

Torbinejad introduced mineral trioxide aggregate (MTA) to dentistry as a root end filling and perforation repair material. It is a modified Portland cement that consists of tri-calcium and di-calcium silicate, tri-calcium aluminate, tetra-calcium aluminoferrite, and bismuth oxide. There are two types—Gray and White MTA—and the difference between them is the absence of iron in white MTA. MTA is a paramount material used in perforation repair, as it meets the ideal properties above [5]. It has excellent biocompatibility, superior sealing ability, and is able to set in the presence of blood contamination [6,7,8]. However, despite its favorable properties, it has a prolonged setting time, is difficult to handle, may discolor, and is washes out easily during an immediate irrigation procedure [9,10].

Bioceramic is a new technology introduced in endodontics as a root repair material and root canal sealer to compensate for standard MTA’s disadvantages. EndoSequence root repair material (ERRM), which Brasseler introduced (ERRM; Brasseler, Savannah, GA) is a nano-bioceramic material composed of calcium silicates, calcium phosphate monobasic, and zirconium oxide without bismuth oxide [11]. This material is hydrophilic, insoluble, and radiopaque. It also is biocompatible [12,13] and has an antibacterial effect [14] attributable to its highly alkaline pH [15]. In addition, it is bioactive and bioresorbable, which allow the material to participate in the dynamic process of bone formation and re-absorption that leads to bone regeneration and facilitates periodontal tissue repair [16].

Different ERRM formulas, both in a low viscosity paste and high viscosity putty, are available in the market to improve their clinical application. Although EndoSequence’s composition is very similar to that of MTA, it has a different setting time. Damas et al. indicated that, although ERRM appeared to set completely after 7 days, it is possible that some remnant material did not set internally [17]. Thus, a new, improved formula of fast-set EndoSequence was introduced, the working time of which is more than 30 min, and the setting time 4 h under normal conditions. 

Different studies have advocated repairing a perforation as soon as possible, even before the completion of root canal treatment, to prevent bacterial contamination [18,19]. While completing endodontic treatment, the repair material is subjected to different irrigants, such as sodium hypochlorite (NaOCl) and chlorhexidine gluconate (CHX), to disinfect the root canal system. However, these irrigants may have unfavorable effects on repair materials. Guneser et al. reported that MTA lost strength when exposed to CHX [20]. The interfacial bond strength between the repair material and dentin wall is indicative of adequate mechanical adhesion to maintain the material’s sealing integrity [21]. However, no studies have evaluated irrigants’ effects on ERRM’s adhesive properties adequately. 

The purpose of this study was to compare different irrigating solutions’ effect (5.25% NaOCl and 2% CHX) on the push-out bond strength of ERRM fast-set putty and regular-set paste. The null hypothesis is that the two do not differ in push-out bond strength after using either 5.25 % NaOCl or 2% CHX irrigating solutions. 

## 2. Materials and Methods 

### 2.1. Specimen’s Preparation for Push-Out Test

King Abdulaziz University ethical committee approved the procedures in this study (# 063-04-18). A total of thirty freshly extracted, single-rooted human teeth was used. The crowns of all teeth were decoronated close to the cementum-enamel junction using a water-cooled diamond bur. Each root was embedded vertically in a rubber mold containing epoxy resin (Vertex Orthoplat; Vertex–Dental, Zeist, Utrecht, The Netherlands). Each tooth’s mid-root was sectioned horizontally into two slices (2.00 ± 0.05 mm thickness) using a microcut machine (TechCut 4™, Precision Low Speed, Rancho Dominguez, CA, USA), and each slice’s canal space was enlarged with Gates Glidden burs (DENTSPLY Maillefer, Ballaigues, Switzerland), sizes 2–6, to achieve a diameter of 1.5 mm. All root canals’ dentin walls were treated with 17% ethylenediaminetetraacetic acid (EDTA: Pulpdent EDTA 17% Solution, Pulpdent Corporation, Watertown, MA, USA) for 3 min, rinsed with distilled water to remove the dentin debris, and then dried with a paper point. The 60 slices prepared were divided randomly into two main groups. The first was filled with premixed ERRM fast-set putty while the second was filled with the premixed ERRM regular-set syringable paste. The material was packed inside the canal space with a plastic instrument, after which the excess material was removed with a scalpel. Specimens were wrapped with moistened gauze, sealed in a container, and placed in an incubator at 37 °C and 100% relative humidity for 3 days until they set completely. 

According to the irrigant used, each group was divided randomly into three subgroups (10 each). Specimens were immersed either in 5.25% NaOCl (first subgroup), 2% CHX (second subgroup), or normal saline (control subgroup) for 30 min. Then, specimens were removed, rinsed with distilled water, and placed in an incubator for 48 h. 

### 2.2. Push-Out Bond Strength Testing

The materials’ dislocation resistance was measured using the push-out strength test with a universal testing machine (Instron, Model 5944 MicroTester Precision Instruments, Norwood, MA, USA). The samples were placed on a metal slab containing a central hole to allow the free motion of a plunger 1.2 mm in diameter, with a constant vertical downward pressure at a speed of 0.5 mm/min. The test was carried out until total bond failure. The maximum force applied to materials at the time of dislodgement was recorded in Newton, while the push-out bond strength was calculated in megapascal (MPa) machine software (Instron Bluehill Universal, Norwood, MA, USA) [22]. 

### 2.3. Failure Mode Analysis

After performing the push-out test, the fractured specimens were evaluated under a stereomicroscope (Meiji Techno Co. Ltd., Tokyo, Japan) at 50× magnification. Each sample was categorized according to one of three failure modes: An adhesive failure that occurred at the dentin–material interface, cohesive failure that occurred within the material, or mixed failure, a combination of the two failure modes. 

### 2.4. Surface Microstructure Analysis

Nine discs of each material 10 mm in diameter and 3 mm thick were prepared. Each was wrapped with moistened gauze and stored in an incubator at 37 °C and 100% relative humidity for 3 days. After they set completely, the discs were immersed in 5.25% NaOCl, 2% CHX, or saline for 30 min. The changes in surface structure after the irrigants’ application were evaluated using a Scanning Electron Microscope (SEM, Quanta 250 Field Emission Gun attached, FEI Company, Eindhoven, The Netherlands).

### 2.5. Fourier Transform Infrared Spectroscopy Analysis

All specimens were subjected to chemical analysis using Fourier Transform Infrared Spectroscopy (FT/IR-6100, Jasco, Tokyo, Japan) to determine the chemical changes that occurred after immersion in different irrigants. The spectra were recorded in the range of 4000–400 cm^−1^ with 1 cm^−1^ resolution. To reduce the noise-to-signal ratio, each spectrum was scanned several times (Ribeiro TJ 2014), and different bands’ changes in location and intensity were recorded. The carbonate/phosphate (CO_3_/PO_4_) ratio of the surface material was calculated to determine each irrigant’s effect on the material constituents. The integrated area under the carbonate (v_3_CO_3_) band at 830–890 cm^−1^ was divided by the integrated area under the phosphate (v_1_v_3_PO_4_) band at 900–1200 cm^−1^, as described in previous studies [23,24,25].

### 2.6. Statistical Analysis

A one-way (ANOVA) and Post-Hoc Tukey tests were used to compare the push-out bond strength of samples of each material after immersion in different irrigants, and the Chi-squared test was used to compare the failure mode between subgroups. The irrigants’ effect on both materials’ carbonate/phosphate ratio also was compared with the student’s t-test (between materials) and one-way ANOVA (between irrigants). *P* was set at 0.05. Data were analyzed using Statistical Package for the Social Sciences (SPSS) version. 16 (SPSS Inc., Chicago, IL, USA).

## 3. Results

### 3.1. Push-Out Bond Strength Testing

**Figure 1:**
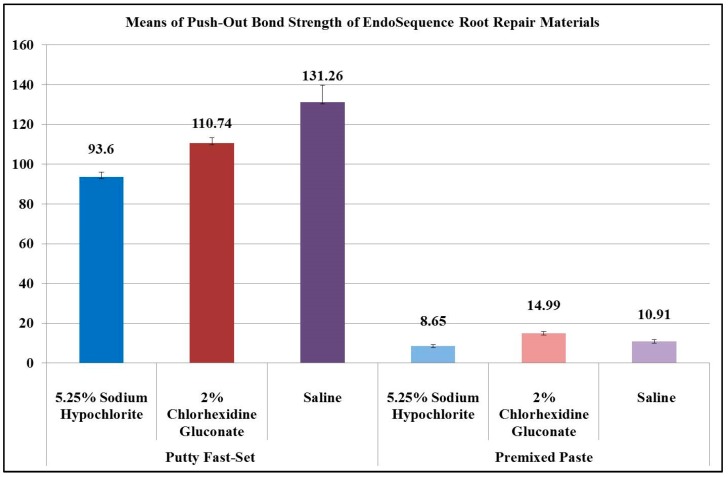
Mean values of experimental groups’ push-out bond strength.

Figure 1 shows the mean values of the experimental groups’ push-out bond strength, in which the EndoSequence fast-set putty showed significantly higher values (*p* = 0.000). The fast-set putty exposed to saline displayed a significantly greater mean push-out bond strength (131.26 ± 26.68 MPa) compared to that exposed to 2% CHX (110.74 ± 8.09 MPa: *p* = 0.024) and 5.25% NaOCl (93.6 ± 7.73 MPa: *p* = 0.000), respectively. There was no significant difference between fast-set putty treated either with 2% CHX or 5.25% NaOCl (*p* = 0.074). The regular-set paste samples exposed to 2% CHX demonstrated a significantly higher mean push-out bond strength (14.99 ± 2.48 MPa) compared to those exposed to 5.25% NaOCl (8.65 ± 2.02 MPa: *p* = 0.000) and saline (10.91 ± 2.88 MPa: *p =* 0.003), respectively. There was no significant difference between the two latter subgroups (*p* = 0.12).

### 3.2. Nature of Failure Mode

**Figure 2:**
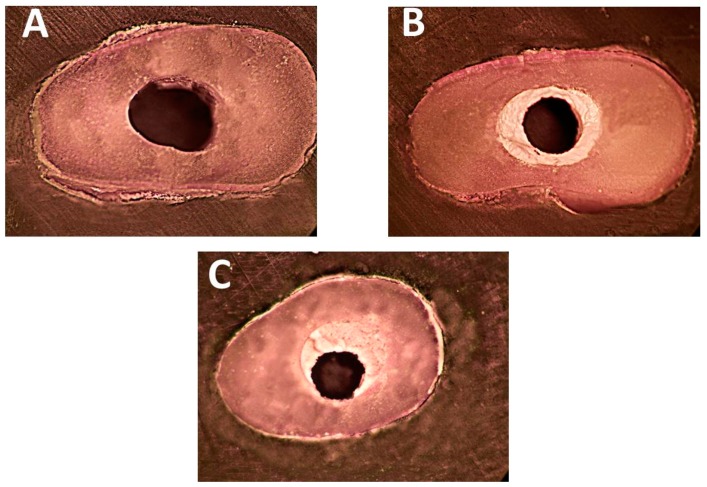
Stereo-photomicrographs of failure modes after push-out test at 50× magnification (**A**) Complete adhesive: the canal is completely clean, (**B**) Complete cohesive: the failure occurs within the EndoSequence material, (**C**) Mixed failure: Remnants of EndoSequence remain inside the canal.

Table 1 illustrates the failure mode distribution among the subgroups investigated. Adhesive and mixed failure modes predominated in specimens filled with EndoSequence fast-set putty (Figure 2A,C) with no significant difference between subgroups regardless of the irrigating solutions used (*p* > 0.05). However, in specimens filled with EndoSequence premixed paste, the mixed and cohesive failure modes predominated (Figure 2B,C) and there were significant differences depending upon the irrigants used (*p* < 0.05). Figure 2 shows the different categories of failure mode determined by stereomicroscopy. 

### 3.3. Surface Microstructure Analysis

**Figure 3:**
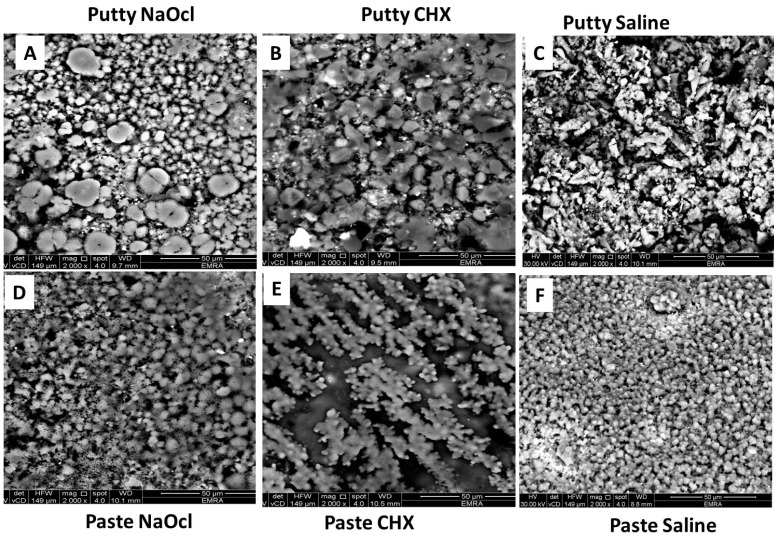
Scanning Electron Microphotographs of surface topography of putty (**A**–**C**), paste (**D**–**F**) root repair materials after immersion in 5.25% sodium hypochlorite, 2% chlorhexidine gluconate, and saline, respectively.

The surface of the ERRM putty showed a biphasic surface morphology under Scanning Electron Microscopy (SEM), in which there were clusters of small, globular particles interrupted by ill-defined, faceted, larger particles after immersion in 5.25% NaOCl (Figure 3A). After immersion in 2% CHX, ill-defined globular particles were packed on the surface (Figure 3B), while the samples immersed in saline showed clusters of large, irregular-shaped particles (Figure 3C).

The surface of ERRM regular-set paste immersed in 5.25% NaOCl (Figure 3D) was covered with aggregates of small particles with a sponge-like structure. After immersion in 2% CHX, the surface was covered with separate agglomerates of small, globular particles with a coral reef structure (Figure 3E), while after immersion in saline, it showed diffused aggregates of fused, small globular particles (Figure 3F).

### 3.4. Fourier Transform Infra-Red Analysis

**Figure 4:**
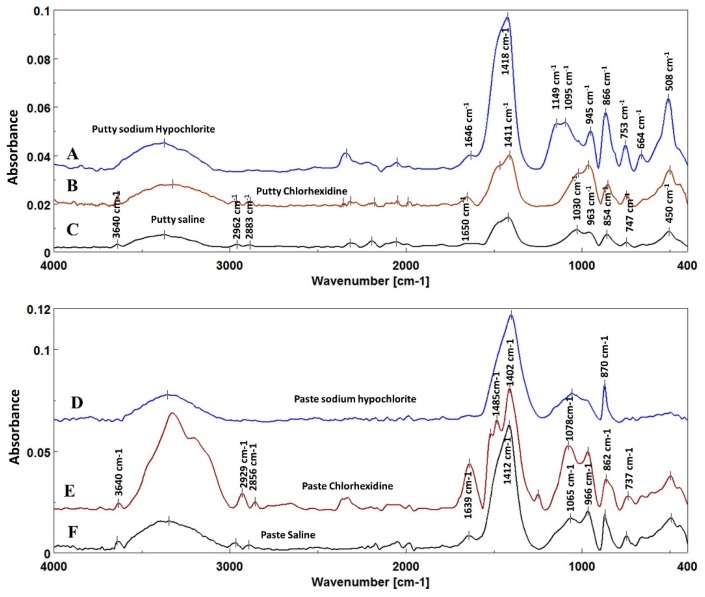
Fourier Transform Infrared spectra of EndoSequence fast-set putty (top A–C) and premixed regular-set paste (bottom D–F) show the changes in different bands after immersion in 5.25 % sodium hypochlorite (A and D, blue) and 2% chlorohexidine gluconate (B and E, brown) compared to that immersed in saline (C and F, black).

Fourier transform Infrared (FTIR) spectra of both materials investigated revealed changes in the organic fillers’ asymmetric CH stretch band at 3000–2800 cm ^−1^ [26,27] and the Portlandite (calcium hydroxide) band at 3640 cm^−1^ [23,28,29] that disappeared after immersion in 5.25% NaOCl (Figure 4A,D), while it humped and shifted at a lower wavenumber after immersion in 2% CHX (Figure 4B,E) compared to that immersed in saline (Figure 4C,F).

Carbonate bands (v_3_CO_3_) at 1500–1400 cm^−1^ and (v_2_CO_3_) at 890–830 cm^−1,^ as well as a phosphate band (v1v3PO_4_) at 1200–900 cm^−1^ [30,31,32] of both fast-set putty and premixed regular-set paste also showed changes in their width and intensity after immersion in different solutions. There was no significant difference in the carbonate/phosphate ratio obtained with fast-set putty versus regular-set paste (*p* > 0.05, Table 2). The samples of both materials immersed in 2% CHX had a significantly lower carbonate/phosphate ratio (0.17 ± 0.01 for fast-set putty and 0.19 ± 0.02 for regular-set paste: *p* = 0.001), followed by materials immersed in 5.25% NaOCl (0.22 ± 0.03 and 0.26 ± 0.04, respectively) and materials immersed in saline (0.23 ± 0.04 and 0.27 ± 0.04 respectively: *p* = 0.84). There was no significant difference between the latter two groups.

## 4. Discussion

One of a perforation repair material’s principal desirable properties is its adhesive property that increases displacement resistance [33], and the push-out bond test is an effective and reliable method to evaluate root repair materials’ adhesive integrity [34,35]. This study focused on evaluating the effect of the irrigants used most widely—5.25% NaOCl and 2% CHX—on the adhesive behavior and push-out bond strength of ERRM of two different formulas: fast-set putty versus regular-set paste. Although the root canal spaces were treated with 17% EDTA for 3 min to increase the dislodgment resistance of the materials investigated based on previous studies’ results [36,37], both materials had different push-out bond strength, and thus, the study’s null hypothesis was rejected. 

In general, the fast-set putty group exhibited significantly greater strength compared to the regular-set paste group, regardless of the type of irrigant used. This finding may be attributable to the materials’ microstructures and grain (particle) size. SEM analysis showed that the fast-set putty had a larger particle size that helped sustain higher loads while the regular-set paste consisted of particle sizes that are too small and thus, can be destroyed easily with low loads as the results demonstrated. Further, it has been reported that EndoSequence putty contains a filler and thickening agent that may increase the core bond strength within the material [38] and resist cohesive failure. 

The different types of failure modes found in the putty versus paste may be attributable to the materials’ consistency. The fast-set putty has a heavy consistency that makes it difficult for it to penetrate dentinal tubules, which leads to easy displacement from the dentin wall under load (adhesive failure). In contrast, the regular-set paste has a consistency that causes it to flow better and has a small particle size that allows the material to penetrate the dentinal tubules easily and resist displacement forces. However, fracture still can occur when the core is fragile (cohesive failure). The results of our study are consistent with those of a previous study that showed that calcium silicate material with small particles that flowed readily permitted the formation of a tag-like structure (monoblock layer) at the dentin/cement interface that was responsible for mechanical bonding, better interlocking, and greater displacement resistance [39].

Our results showed that the push-out bond strength increased significantly after immersion in saline followed by 2% CHX in the putty groups, while the paste groups showed that 2% CHX followed by saline increased the push-out bond strength. SEM analysis demonstrated that the addition of CHX solution to the fast-set putty did not decrease the particle size, but caused erosion of its border instead that led to an ill-defined globular structure. However, in the regular-set paste specimens, chlorhexidine increased the strength significantly, as it collected the small particles into diffused aggregates of fused, small, globular particles with a coral reef structure that may resist fracture. This finding was contrary to that of previous studies that showed that 2% CHX reduced calcium silicate materials’ (MTA) push-out strength [20].

According to FTIR analysis, when the material was immersed either in saline and/or CHX, a series of reactions took place on the surface of the bioceramic root repair material, in which inorganic mineral bands of Portlandite (calcium hydroxide) were present at 3640 cm^−1^ [23,28,29], and the width and intensity of the carbonate bands at 1500–1400 cm^−1^ and phosphate bands at 1200–900 cm^−1^ increased [29] in the spectra of material immersed either in saline and/or CHX, which demonstrates their greater surface fracture resistance under load. The reduction of the carbonate/phosphate ratio in material immersed in CHX solution promotes the formation of calcium phosphate crystals that contribute to excellent cohesive bond strength on the material’s surface. 

FTIR spectra of both materials immersed in 5.25% NaOCl showed that the Portlandite (calcium hydroxide) band dipped at 3640 cm^−1^ [23,28,29] compared with spectra of other solutions. This finding may be attributed to rapid consumption and dissociation of calcium hydroxide when the material surface contacts NaOCl and is responsible for the decreased mineral element of the material surface and reduced fracture resistance. 

Thus, 5.25% NaOCl reduced the push-out bond strength markedly in both materials investigated. Although both materials’ particle sizes were relatively larger compared with those immersed in saline, they appeared to be separated from each other by small gaps. It has been suggested that organic filler is added to EndoSequence to allow inorganic elements to bind and form a cohesive mass [38,39]. Their FTIR spectra showed that the organic band disappeared at 3000–2800 cm^−1^ compared with the other subgroups’ spectra. 

This finding may be attributable to NaOCl’s dissolving effect on the organic filler, which causes loss of the inorganic minerals on the material’s surface and reduced surface fracture resistance. These results contradicted those in Alsubait’s study, which showed that NaOCl improved premixed EndoSequence paste’s bond strength [39]. 

## 5. Conclusions

Under the circumstances of this study, we can conclude that after repairing root perforation using either fast-set putty or regular-set paste EndoSequence, the use of 5.25% NaOCl reduced the displacement resistance markedly when subjected to condensation force. Therefore, clinicians are advised to take care to prevent NaOCl from contacting the ERRM in a single visit treatment, and perhaps postpone the root canal treatment to the next visit after using ERRM to ensure that it is set completely before any irrigation solution is used.

## Figures and Tables

**Table 1:** Distribution of failure modes among the experimental subgroups: adhesive failure occurs at the dentin–material interface, cohesive failure occurs within the material, and mixed failure is a combination of the two failure modes.

	Failure Mode	Cohesive of 10	Adhesive of 10	Mixed of 10	*p*-Value
Subgroups					
Putty 5.25 % Sodium Hypochlorite		1 (10%)	4 (40%)	5 (50%)	0.150
Putty 2% Chlorhexidine Gluconate		3 (30%)	2 (20%)	5 (50%)	0.150
Putty Saline		2 (20%)	6 (60%)	2 (20%)	0.905
Paste 5.25 % Sodium Hypochlorite		1 (10%)	1 (10%)	8 (80%)	0.007
Paste 2% Chlorhexidine Gluconate		6 (60%)	0 (0%)	4 (40%)	0.007
Paste Saline		3(30%)	1(10%)	6 (60%)	0.000

**Table 2:** Means ± standard deviations of EndoSequence (fast-set putty and regular-set paste) carbonate/phosphate ratio after immersion in different irrigating solutions.

	Materials	Putty Fast-Set Means ± SD	Paste Regular-Set Means ± SD	*t*-Test
Irrigants				
5.25% Sodium Hypochlorite		0.22 ± 0.03	0.26 ± 0.04	2.77, *p* = 0.11
2% Chlorhexidine Gluconate		0.17 ± 0.01	0.19 ± 0.02	1.77, *p* = 0.20
Saline		0.23 ± 0.04	0.27 ± 0.04	2.05, *p* = 0.95
ANOVA		F_df_ = 9.35 *p* = 0.001	F_df_ = 9.32 *p* = 0.001	-
